# [Corrigendum] Individualized therapy based on the combination of mini-PDX and NGS for a patient with metastatic AFP-producing and HER-2 amplified gastric cancer

**DOI:** 10.3892/ol.2026.15913

**Published:** 2026-10-21

**Authors:** Xiaodan Zhu, Xiaoqing Xu, Baonan Zhang, Yun Dong, Shixia Gong, Tingjie Gong, Feifei Zhang, Chunhui Jin

Oncol Lett 24: 411, 2022; DOI: 10.3892/ol.2022.13531

Following the publication of the above paper, it was drawn to the Editor's attention by a concerned reader that the central claims of this case report appeared to be somewhat overstated relative to the evidence presented. The entire study was based on a single patient (n=1), which is insufficient to establish any predictive validity for the mini-PDX platform that was employed. Moreover, the reader considered that the study design did not permit a determination of whether the observed remission of the patient resulted from treatment with irinotecan + S-1, trastuzumab, or the known benefit of HER2-targeted therapy itself.

In addition, according to the “*Mini-PDX assay”* subsection of the Materials and methods section, concerns were raised regarding design aspects of this part of the study. A total of 8 female severe combined immunodeficiency mice were randomized to four reported groups, implying that 2 mice were allocated to each treatment group. Each mouse was reported to have received 6 capsules at the same time, and each regimen was tested in 2 mice; the final results were calculated as the average of 6 repeated trials, and in the “*Statistical analysis*” subsection, the authors stated that one-way ANOVA was used to evaluate differences among groups. Given that the ANOVA statistical test requires independent samples, the capsules implanted within the same mouse shared the identical physiological environment, drug metabolism, and systemic responses, indicating that they were technical replicates, not biological replicates. It was suggested that the authors should clarify exactly what “n” represented in the error bars and statistical tests shown for Fig. 5; if n=6 were to refer to capsules, rather than independent mice, using an ANOVA test for a biological sample size of n=2 may have been inappropriate.

The authors have now responded to the concerns of the reader, and wish to make the following amendments to/clarifications of the relevant data, and the interpretation thereof, in their published paper: First, the authors wish to confirm that they did not intend to claim that the observed remission was exclusively due to the mini-PDX-guided chemotherapy. They fully accept that their study is a single case report with inherent limitations, and mentioned at the end of the Abstract that “the proposed technique requires further research to confirm its potential in the individualized treatment of patients with refractory malignancies”. In the Discussion section, on p. 7, left-hand column, line 4, after describing the satisfactory outcomes and that complete remission was achieved using the combination of irinotecan and S-1 with trastuzumab, the authors wish to add the following sentence: “**The authors wish to explicitly state that this single-patient case does not establish the predictive validity of the mini-PDX platform, and that the clinical remission cannot be attributed specifically to the mini-PDX-selected chemotherapy, since trastuzumab was administered concurrently.**”

The authors also acknowledged the concern regarding the unit of analysis that they had employed for the statistical treatments. The text in the “*Statistical analysis of mini-PDX*” subsection of the Materials and methods has therefore been revised to read as follows: “**The biological sample size was n=2 mice per group for the experiments performed with the mini-PDX platform. It should be noted that the main goal of the present study was to offer descriptive details in order to generate hypotheses that would warrant more refined exploration in future studies, with suitable statistical analyses.**”

The authors also agree that identifying body-weight stability in two mice would be insufficient to establish a margin of safety. Therefore, the text in the Results section towards the end of the “Mini-PDX assay” subsection on p. 3, should be revised to read as follows: “**Furthermore, no marked reduction in the body weight of the mice was observed in any group during the experiment (Fig. 5B). It should be noted that body weight was monitored only as an observational parameter in these experiments, and it was not possible to establish a margin of safe**ty.” In line with these changes, as shown on the next page, Fig. 5 and its legend, showing the response of the mini-PDX assay to various chemotherapy regimens and the change of body weight during drug delivery, have also been revised to reflect that these results are presented only descriptively. In addition, the P-values and statistical indicators have been removed from Fig. 5A and the legend.

The authors hope that these textual amendments/clarifications will satisfy the concerns of the reader, and believe that these changes/explanations communicate more effectively the descriptive and hypothesis-generating nature of the study. They are grateful to the Editor of *Oncology* *Letters* for allowing them this opportunity to respond to the reader's concerns, and all the authors agree to the publication of this corrigendum, while apologizing for any inconvenience caused.

## Figures and Tables

**Figure 5.**
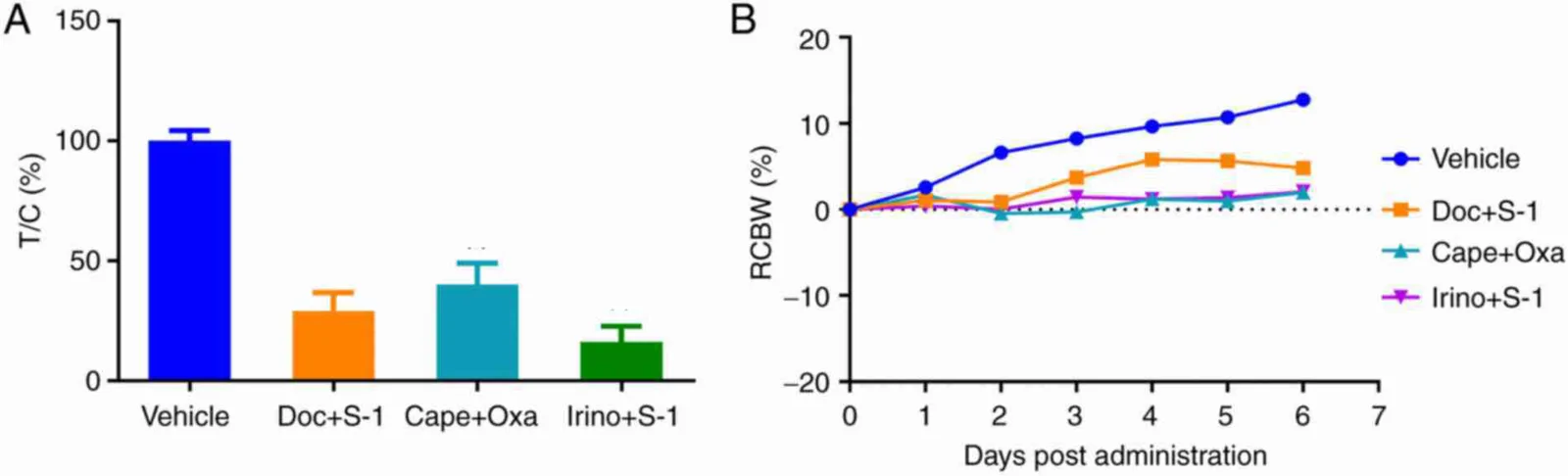
Response of the mini-PDX to various chemotherapy regimens and the change of body weight during drug delivery. (A) The proliferation of tumor cells in mini-PDX implants was observed in mice treated with vehicle, Doc + S-1, Cape + Oxa and Irino + S-1. The drug treatment began 1 day after tumor cell implantation. The tumor cell proliferation rate relative to that of the control group was calculated after the 7-day treatment. (B) The change in body weight of the mice was monitored. Throughout the experiment, no marked reduction in the weight of mice in each group was detected. **It should be noted that body weight was monitored only as an observational parameter in these experiments, and it was not possible to establish a margin of safety**. T, tumor; C, control; Doc, docetaxel; Cape, capecitabine; Oxa, oxaliplatin; Irino, irinotecan; S-1, tegafur-gimeracil-oteracil (tegio); RCBW, relative change in body weight.

